# Muscle Contractile Characteristics During Exhaustive Dynamic Exercise and Recovery

**DOI:** 10.3389/fphys.2021.660099

**Published:** 2021-07-02

**Authors:** Fabrice Rannou, Lars Nybo, Janni Enghave Andersen, Nikolai B. Nordsborg

**Affiliations:** ^1^Department of Sport Medicine and Functional Explorations-ASMS, CRNH, CHU Clermont-Ferrand, Clermont-Ferrand, France; ^2^Department of Nutrition, Exercise and Sports, University of Copenhagen, Copenhagen, Denmark

**Keywords:** dynamic exercise, intramuscular fatigue, electromechanical delay, rates of force development and relaxation, recovery

## Abstract

Our aim was to provide an *in vivo* assessment of human muscle twitch characteristics during and following an exhaustive dynamic exercise to explore temporal alterations of the rate of force development (RFD) and relaxation (RFR). Eleven healthy participants (mean age ± SD: 24 ± 3 years) completed a dynamic knee-extensor exercise in randomized order at three different intensities, eliciting exhaustion after ∼9 min (56 ± 10 W), ∼6 min (60 ± 10 W), and ∼4 min (63 ± 10 W), in addition to a low-intensity (28 ± 5 W) bout. In a novel setup, an electrical doublet stimulation of m. vastus lateralis was applied during exercise (every 30 s) and recovery for frequent evaluation of key contractile properties (maximal force, RFD, RFR, and electromechanical delay) in addition to M-wave characteristics. RFD and RFR remained stable throughout the low-intensity trial but declined in all exhaustive trials to reach a similar level of ∼40% of pre-exercise values at task failure but with the exponential decay augmented by intensity. Following exhaustion, there was a fast initial recovery of RFD and RFR to ∼80% of pre-exercise values within 1 min, followed by a longer suppression at this level. The M-wave characteristics remained unchanged during all trials. In conclusion, this is the first study to quantify the intensity-dependent alterations of RFD and RFR during and after exhaustive dynamic exercise in humans. A hypothesized reduction and fast reversion of RFD was confirmed, and a surprising compromised RFR is reported. The present unique experimental approach allows for novel insight to exercise-induced alterations in human muscle contractile properties which is relevant in health and disease.

## Introduction

Skeletal muscle maximal force-generating capacity is gradually lost with exhaustive electrical stimulations (Reid, 1927), repeated voluntary submaximal and maximal isometric contractions in humans (Bigland-Ritchie et al., 1986; Burnley, 2009; Burnley et al., 2012), and sustained maximal voluntary contraction (Place et al., 2007; Carroll et al., 2017; Rodriguez-Falces and Place, 2017). The force-generating capacity is also reduced during/after a dynamic isolated exercise (e.g., dynamic knee-extension; Li et al., 2002; Amann et al., 2013; Froyd et al., 2013; Casuso et al., 2014) and a whole-body exercise (e.g., cycling; Millet et al., 2003; Martin et al., 2004; Ducrocq et al., 2017). Intramuscular fatigue development is highly dependent on exercise intensity, especially around critical torque during isometric exercises (Burnley, 2009), and critical power (CP), where muscle creatine phosphate, lactate, and pH remain stable during exercise below but not above CP (Vanhatalo et al., 2016). Moreover, the decrease of normalized compound muscle action potential (M-wave amplitude) after exercise at intensities causing a task failure after 2–14 min is similar and correlates with the metabolic perturbations (Black et al., 2017). In line with the observed metabolic and neural observations, the mechanical properties most often described as maximal voluntary force after exhaustion also demonstrate a clear dependence on exercise intensity (Brownstein et al., 2021). However, there is a need for addressing alterations in muscle mechanical properties during exercise, which is the aim of the present study. By using a novel model allowing electrical stimulations during exercise for investigating human peripheral muscle fatigue, defined as exercise-induced loss of force-generating capacity, we recently extended these findings to repeated dynamic contractions in humans and demonstrated an intensity-dependent gradual reduction of maximal twitch force (*F*_tw_) during an exhaustive exercise (Rannou et al., 2019). Importantly, *F*_tw_ reduction occurs rapidly within the first few minutes of a ∼6-min exercise period to task failure, and the loss of *F*_tw_-generating capacity is highly dependent on intensity changes.

Numerous mechanisms can cause the impairment of the F_tw_-generating capacity, including impaired excitation–contraction coupling and Ca^2+^ handling as extensively reviewed (Palmer et al., 1991; Allen et al., 2008; Fitts, 2008; Debold et al., 2016). It has been known for more than a century that skeletal muscle fatigue compromises not only *F*_tw_ but also the rate of force development (RFD) and relaxation (RFR) as well as the action potential propagation (Fletcher, 1902; Westerblad and Lännergren, 1991; Allen et al., 2008; Fitts, 2008). Based on *in vitro* findings, a plausible mechanistic explanation for the compromised *F*_tw_ and RFD is a gradual impairment of Ca^2+^ release rate (Cheng et al., 2018; Olsson et al., 2020) secondary to inorganic phosphate (P_i_)-induced sarcoplasmic reticulum (SR) Ca^2+^ precipitation and ryanodine receptor 1 (RyR1) inhibition (Allen et al., 2008), even though other possibilities exist, including metabolic acidosis (Fitts, 2008). In humans, a marked reduction of ∼40% in maximal twitch RFD occurs as a consequence of an intense exhaustive exercise in humans (Krüger et al., 2019), and we have previously demonstrated the twitch RFD to be compromised during an exhaustive dynamic exercise (Casuso et al., 2014), but without addressing the impact of intensity.

As an additional measure, RFR is prolonged in fatigued isolated muscle preparations due to slowed Ca^2+^ reuptake as well as impaired cross-bridge mechanisms (Allen et al., 1995; Li et al., 2002). Importantly, the marked slowing of relaxation has also been observed in human muscles subjected to fatiguing electrical stimulation (Neyroud et al., 2016) as well as during exercise (Casuso et al., 2014), although very little is known about the effect of intensity. As for RFD, the determination of the intensity-dependent changes in RFR with fatigue development in humans is a feasible way to gain insight to possible underlying mechanisms.

In addition to muscular contractile parameters determined from electrically induced twitches, electromechanical delay (EMD) and M-wave properties yield information about muscular electro-mechanical function. EMD reflects synaptic transmission, action potential propagation along the sarcolemma, excitation–contraction coupling, and transmission of force along passive series-elastic elements (Blackburn et al., 2009), whereas M-wave properties are commonly used as an indirect marker of membrane excitability (Rodriguez-Falces and Place, 2017). Since impaired membrane excitability secondary to extracellular K^+^ accumulation has frequently been proposed as causative for the development of muscle fiber fatigue during exhaustive exercise (Hostrup and Bangsbo, 2017), we also quantified these parameters. The temporal pattern and intensity dependence of excitability changes will provide mechanistic insight to the possible role of developing inexcitability for human muscle fatigue manifestation.

The time-course of muscle fatigue recovery is also important for daily activities and sports involving intermittent intense exercise. During recovery, exercise-induced impairment of muscle contractile properties has to be reversed to restore the ability of the muscle to produce force. Previous studies have highlighted that the time-lag between task failure and the measurement of muscle contractile parameters is a crucial issue to fully investigate recovery (Froyd et al., 2013; Carroll et al., 2017). However, from a methodological perspective, it is a challenge to investigate the mechanisms responsible for fatigue development *in vivo* as well as their recovery time-course. In the present paper, we aim to determine the contractile and electrical characteristics of the human skeletal muscle with a high temporal resolution. This information will reveal the likely underlying causes of human fatigue development within the muscle itself during exhaustive dynamic exercise. Therefore, we determined the twitch characteristics and their dependencies on exercise intensity.

The study hypothesis was that the intensity-dependent loss of muscle *F*_tw_-generating capacity during continuous exhaustive exercise at constant load co-occurs with a compromised RFD, RFR, EMD, and M-wave (i.e., “intratwitch indexes of contractile properties”).

## Materials and Methods

### Participants

Eleven healthy, physically active individuals (seven males, four females; age, 24 ± 3 years; body mass index, 22 ± 2 kg/m^2^) were recruited for this investigation. To be eligible for participation, the subjects were required to be free from any knee or neuromuscular disorders. All the participants provided informed written consent to this study, which was approved by the Copenhagen and Frederiksberg Ethics Committee (H-16035688) and complied with the latest (2013) Declaration of Helsinki amendment.

### Procedures

**FIGURE 1:**
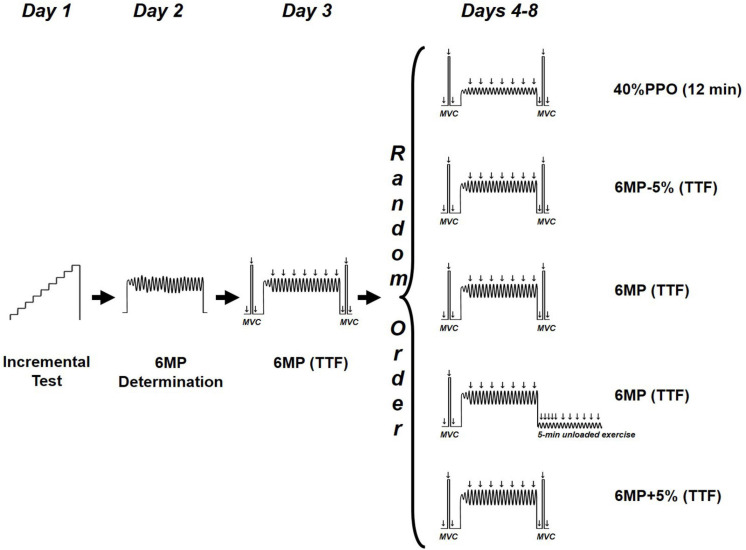
Study design. TTF, time to task failure; 6MP, power eliciting task failure within 6 min; PPO, peak power output.

The subjects completed eight testing sessions of a single-leg knee-extension exercise (Figure 1) on a modified Krogh cycle ergometer (Andersen et al., 1985). This model allows a one-legged knee-extension exercise in dynamic condition, with a knee angular movement between 90° and 170°, as well as in isometric condition at 90° knee angle. Before each experimental trial, the subjects were asked to refrain from strenuous exercise for at least 24 h and the consumption of caffeine or alcohol on test days. The trials were separated by a minimum of 2 days and a maximum of 7 days (3.6 ± 2.3 days, mean ± SD) and were conducted at the same time of the day. The study period lasted up to 24.5 ± 6.5 days for the volunteers.

#### Pre-experimental Procedures

The subjects were seated upright on a chair of the modified Krogh cycle at a 90° thigh-to-trunk angle, with their shoulder and pelvis stabilized by belts.

All testing sessions began with a standardized 5-min warm-up composed of a single-leg, dynamic knee-extensor exercise (males = 18 W; females = 12 W) at 60 rpm. During the first session, a progressive power-increment test was applied to determine the peak power output (PPO). After warm-up, the women and men exercised for 1 min at 30 and 36 W, respectively, followed by a stepwise increase in workload of 6 W × min^–1^ until volitional exhaustion (i.e., inability to sustain the required kicking rate of 60 rpm). In the second session, we sought to estimate the workload likely to exhaust the participants in ∼6 min (“6-min power,” 6MP). The duration of ∼6 min for the exhaustive exercise was chosen to allow disturbance of ion homeostasis during the initial 90 s, i.e., gradual reduction of muscle pH and phosphocreatine and increase of P_i_ (Jones et al., 2008; Burnley et al., 2010). After warm-up, the applied load was set at 85% PPO (with a cadence of 60 RPM), and the subjects were instructed to perform as many kicks as possible within 6 min (Rannou et al., 2019). By averaging the 6-min cadence, the estimated 6-min power output was calculated according to the following formula: 6MP (watt) = 85% PPO (watt) × (kick number in 6 min/360). On day 3, the subjects exercised at 6MP while electrical stimulations were delivered in order to validate the time to task failure would be ∼5–7 min during the experimental days.

#### Experimental Procedures

From days 4 to 8, the subjects performed five randomly administrated trials at either an exercise intensity of 40% PPO for 12 min or four high-intensity exercises until task failure at 6MP-5%, 6MP+5%, and 6MP. The failure criterion for the exhaustive dynamic exercise was an RPM < 57 for five consecutive seconds in spite of strong and standardized verbal encouragement.

Electrical stimulation was delivered *via* doublets in order to optimize the signal-to-noise ratio (i.e., obtain a higher force response) for RFD, RFR, and EMD determination. Double electrical stimuli (200-μs duration and 10-ms interval) was applied transcutaneously to the right m. vastus lateralis using a constant-current stimulator (DS7A Digitimer Ltd., Welwyn Garden City, United Kingdom) *via* self-adhesive surface electrodes of the same size (5 × 9 cm^2^; PALS platinum, Axelgaard, Lystrup, Denmark) placed on a line between the anterior superior iliac spine and the middle of the upper border of the patella. The upper electrode (cathode) was placed at one-third and the lower electrode (anode) at two-thirds of the distance between these two points. Before each experiment, stimulus optimization was performed at rest by slowly increasing the intensity in steps of 20 mA until no further increase of force response and M-wave peak-to-peak amplitude resulted (Place et al., 2007; Burnley, 2009; Froyd et al., 2013). The experimental stimulation intensity was set at 20% higher to ensure supra-maximal stimulation throughout the test (Neyroud et al., 2016; Rodriguez-Falces and Place, 2017).

### Pre- and Post-MVC Twitch Measurements

After a 5-min warm-up (baseline) and after 4 min of unloaded exercise (Pre), unpotentiated and potentiated twitches were elicited 2 s before and 2 s after a maximal voluntary contraction (MVC) lasting 4 s. Verbal encouragement was provided throughout MVCs. This procedure (unpotentiated twitch, MVC, and potentiated twitch) was repeated three times, separated by a 1-min resting period. The reported values for the unpotentiated and potentiated twitches at baseline and Pre are the mean of the three measurements. At 30 s after the four target exercises (40%PPO, 6MP-5%, 6MP, and 6MP+5%; Figure 1), another set of unpotentiated twitch, MVC, and potentiated twitch was collected (Post).

### M-Wave Recordings

Compound muscle action potential (M-wave) of the right m. vastus lateralis was recorded using bipolar surface electrodes (2 × 1 cm^2^) placed 2 cm apart over the belly of the muscle in the direction of the muscle fiber orientation. The skin was shaved, lightly rubbed with fine sandpaper, and cleaned before the placement of the electrodes. A reference electrode was placed over the ipsilateral patella. The signal was amplified, sampled at 1 kHz, filtered (Grass amplifier, AD instruments Powerlab, Warwick, United States), and analyzed offline (Labview, National Instruments, Texas, United States). The electrode location was marked with indelible and toxic-free ink at the end of each session to achieve repeatable placement between trials.

### Recordings During Dynamic Exercise

**FIGURE 2:**
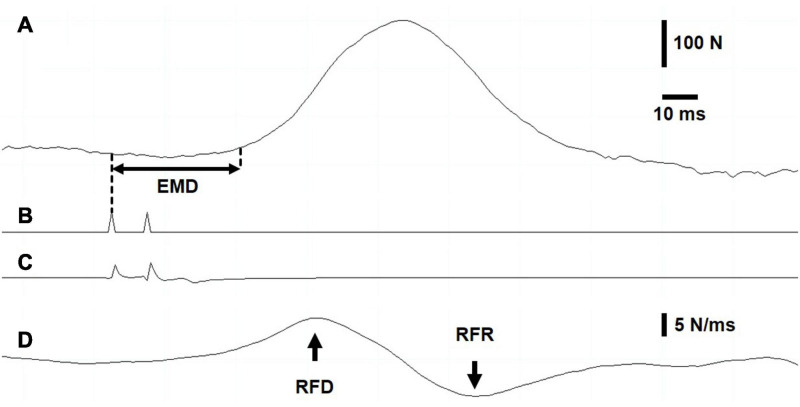
Intratwitch indices measurement during dynamic exercise. Screenshot recording. **(A)** Force recording. The electromechanical delay was measured as the interval between the first electrical stimulation **(B)** of the doublet and the onset of force production, defined as 5% of evoked peak force. **(C)** Stimulation artifact on vastus lateralis electromyogram. **(D)** The maximal rates of force development and relaxation were determined as the highest value and the lowest value, respectively, of the first derivative of the force signal.

During dynamic exercise, stimulation was delivered using a constant-current stimulator (maximum voltage, 400 V; Digitimer DS7A, Hertfordshire, United Kingdom) controlled *via* an automated trigger system (DG2A, Digitimer Ltd., Welwyn Garden City, United Kingdom) (Casuso et al., 2014; Rannou et al., 2019). Briefly, electrical stimulation was delivered as doublets (2 × 200 μs separated by 10 ms) every 30 s in the passive knee-flexion phase (90° knee flexion and 90° hip flexion angle), which allowed a precise quantification of RFD and RFR (Figure 2). Moreover, to study the M-wave properties, a single twitch was elicited (297 ± 77 mA) at the beginning (15 s, start exercise) and at the end of the target intensity exercises (12 min for 40%PPO and at task failure for 6MP-5%, 6MP, and 6MP+5%; end exercise). The characteristics (amplitude, duration, and area) of M-wave first and second phases, as well as the whole M-wave, were measured as recently described by Rodriguez-Falces and Place (2017).

### Recovery During Unloaded Exercise

Following one 6MP exercise (Figure 1), the load was removed, and the subjects were instructed to maintain a 60-RPM cadence (unloaded exercise). After task failure, electrical stimulations were delivered at 10, 20, 30, 45, 60, and 90 s and every 30 s until 5 min after task failure to monitor the recovery of force and intratwitch indices.

### Data Analysis

Force recordings were sampled at 1 kHz (Model 615, Tedea-Huntleigh Electronics, United Kingdom) and processed offline using customized programs written with LabChart software (version 8.1.9, National Instruments, Austin, Texas, United States). The contractile twitch properties were measured from the evoked doublets during dynamic exercise as well as under isometric conditions (baseline, Pre, and Post). Figure 2 displays a screen capture of the representative data (Figure 2A, force recording; Figure 2B, signal from the train/delay generator; Figure 2C, vastus lateralis (VL) electromyogram; Figure 2D, first derivative of the force signal). EMD was determined as the time elapsed between the first trigger signal and the onset of force development (Figure 2A), defined as an increase to 5% of the electrically evoked twitch force (Blackburn et al., 2009). The first derivative of the force signal (Figure 2D) was computed to obtain the peak rate of evoked-twitch force development (highest value; RFD) and relaxation (lowest value; RFR). To compare EMD, RFD, and RFR between subjects during the dynamic target exercise intensities and recovery, the data were normalized to a mean value during unloaded exercise.

### Statistics

The minimum sample size calculation was based on pilot studies demonstrating a mean ± SD RFD of 40 ± 10% at task failure and was determined (GLIMMPSE software) using a type I error probability (α) set at 0.05 and a type II error probability (β) set at 0.2 (power, 80%). The calculated sample size was 10, and 11 subjects were recruited for the present study. The data were analyzed using SPSS V.25 (IBM, Armonk, NY, United States). A two-factorial linear mixed model, appropriate for repeated-measures data, was applied. Intensity, time, and their interaction (intensity × time) were included in the model as fixed effects, and the subject was defined as a random effect. The significant main effects were further examined using Holm–Sidak correction for multiple comparisons. Data are reported as mean ± SD, unless otherwise stated. Statistical significance was accepted at *P* < 0.05.

## Results

### Unloaded Exercise

During unloaded dynamic exercise, the within-subject coefficient of variability for RFD and RFR across four intensities was 5.9 ± 4.9 and 6.9 ± 3.8%, respectively. RFD during unloaded dynamic exercise was 9.8 ± 2.0 N/ms, a value approximately two- to threefold higher than measured under isometric condition during unpotentiated and potentiated twitches (3.4 ± 0.8 and 4.2 ± 1.0 N/ms; Table 1). Similarly, RFR during unloaded exercise (7.5 ± 2.4 N/ms) was approximately three- to fourfold higher compared with twitches determined under isometric condition before loaded exercise (baseline and Pre; Table 1). The RFD and RFR of isometric unpotentiated and potentiated evoked twitches were unaffected by unloaded exercise (baseline vs. Pre; Table 1).

**TABLE 1:** Intratwitch indexes of contractile properties during unpotentiated and potentiated twitches at baseline, before (Pre), and 30 s after (Post) exercise.

**Exercise intensity**	**40% PPO**	**6MP-5%**	**6MP**	**6MP+5%**
**Unpotentiated twitch**				
***Electromechanical delay (ms)***				
Baseline	25.9 ± 2.4	25.9 ± 3.4	25.8 ± 2.7	26.2 ± 3.2
Pre	25.7 ± 2.3	25.7 ± 3.6	25.8 ± 3.5	25.7 ± 3.5
Post	25.8 ± 3.3	26.2 ± 2.8	25.5 ± 2.2	26.3 ± 3.6
***Rate of force development (N/ms)***				
Baseline	3.4 ± 0.7	3.3 ± 0.9	3.3 ± 0.7	3.6 ± 0.7
Pre	3.5 ± 0.9	3.4 ± 0.9	3.5 ± 0.8	3.5 ± 0.6
Post	3.3 ± 0.8	2.2 ± 0.7^a,b^	2.2 ± 0.8^a,b^	2.3 ± 0.8^a,b^
***Rate of force relaxation (N/ms)***				
Baseline	1.6 ± 0.5	1.7 ± 0.8	1.6 ± 0.5	1.6 ± 0.4
Pre	1.7 ± 0.6	1.7 ± 0.6	1.6 ± 0.5	1.6 ± 0.4
Post	1.7 ± 0.5	1.0 ± 0.2^a,b^	0.9 ± 0.4^a,b^	1.0 ± 0.4^a,b^
**Potentiated twitch**				
***Electromechanical delay (ms)***				
Baseline	23.8 ± 1.7	24.0 ± 3.8	23.3 ± 2.4	24.3 ± 3.5
Pre	23.4 ± 2.3	23.2 ± 3.4	23.1 ± 2.9	23.6 ± 2.9
Post	23.7 ± 3.0	25.6 ± 2.9^b,c^	26.0 ± 3.4^a,b^	26.1 ± 2.5^b,c^
***Rate of force development (N/ms)***				
Baseline	4.3 ± 1.1	4.1 ± 1.2	4.2 ± 1.0	4.4 ± 1.0
Pre	4.2 ± 0.9	4.2 ± 1.1	4.5 ± 1.3	4.4 ± 0.9
Post	4.0 ± 1.2	2.3 ± 0.5^a,b^	2.5 ± 0.8^a,b^	2.5 ± 0.4^a,b^
***Rate of force relaxation (N/ms)***				
Baseline	2.0 ± 0.6	2.0 ± 0.8	2.1 ± 0.6	2.1 ± 0.6
Pre	2.1 ± 0.6	2.1 ± 0.7	2.1 ± 0.6	2.1 ± 0.6
Post	2.1 ± 0.6	1.2 ± 0.3^a,b^	1.0 ± 0.4^a,b^	1.0 ± 0.3^a,b^

*Unpotentiated and potentiated twitches recorded before and after a 5-s maximal isometric contraction force. The recordings were completed after warm-up (baseline), prior to (Pre), and after (Post) the dynamic exercise task at a target intensity. The data are reported as mean ± SD. The data were analyzed using a repeated-measures linear mixed model, followed by Holm–Sidak corrected post hoc test. ^*a*^A difference from baseline and Pre within the same exercise task (P < 0.05). ^*b*^A difference from the 40% PPO test at the same time point (P < 0.04). ^*c*^A difference from Pre within the same exercise task (P < 0.004).*

### Exercise Intensity, Rate of Force Development, and Relaxation

**FIGURE 3:**
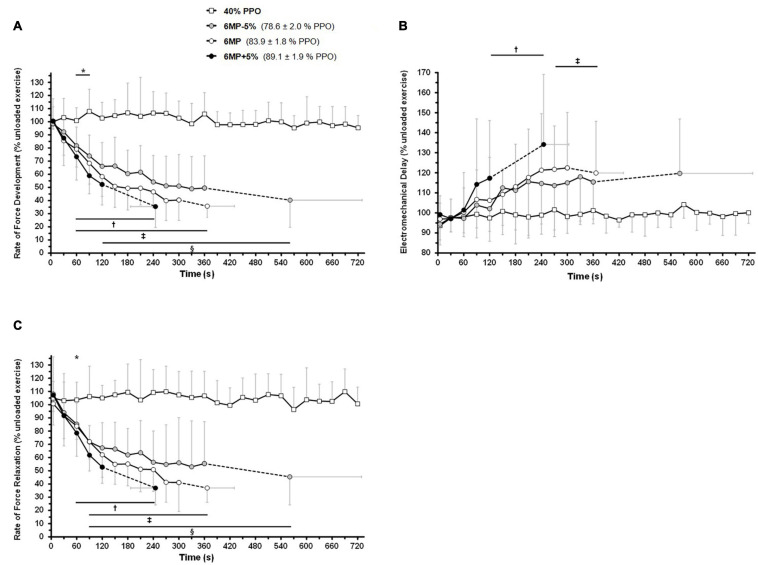
Intensity-dependent effects of dynamic exercise on the rate of force development, relaxation, and electromechanical delay. The participants (*n* = 11) completed 12 min of non-exhaustive, one-legged knee-extension exercise at 40% of peak power output (40% peak power output, PPO) as well as three exhaustive exercise trials at the maximal mean power sustainable for 6 min (6MP) as well as at intensities 5% below (6MP-5%) and above (6MP+5%). M. quadriceps twitches were elicited by a 100-Hz double stimulation delivered in the passive knee-flexion phase at 5 s and subsequently every 30 s of exercise. The first derivative of the force signal was computed to calculate the maximum contraction **(A)** and relaxation **(C)** rates (rate of force development, rate of force relaxation; see section “Materials and Methods”). **(B)** Electromechanical delay. The results are mean ± SD. The data were analyzed taking into account all time points using a repeated-measures linear mixed model, followed by Holm–Sidak corrected *post hoc* test. * 40% PPO is significantly different from 6MP-5% and 6MP at the same time point (intensity effect, *P* < 0.05). ^†^6MP+5%; ^‡^6MP; and ^§^6MP-5% differ significantly from the first time point (5 s) at the same intensity (time effect, *P* < 0.05) and from 40% PPO at the same time point (intensity effect, *P* < 0.05).

The linear mixed-model analysis showed significant effects of time (*P* < 0.001), intensity (*P* < 0.001), and intensity × time interaction (*P* < 0.001) for RFD and RFR throughout dynamic exercise (Figure 3). Regarding time effect (comparison with the first time point, 5 s), RFD decreased during the three exhaustive trials, whereas no changes were observed during 40%PPO (*P* = 1). RFD was decreased after 120 s of exercise during 6MP-5% (*P* = 0.034), while the decline was apparent at 60 s of exercise during 6MP (*P* = 0.013) and 6MP+5% (*P* = 0.011). Compared with 40%PPO (intensity effect), RFD was significantly decreased from 60 s of exercise during 6MP-5% (*P* = 0.003), 6MP (*P* = 0.002), and 6MP+5% (*P* = 0.001). At task failure, the RFD reduction was not significantly different (*P* > 0.19, intensity effect) between the three high-intensity exercise bouts (6MP-5%: 40 ± 21%, 6MP: 36 ± 9%, and 6MP+5%: 35 ± 16%).

From 60 s of exercise, RFR was significantly decreased during 6MP-5% (*P* = 0.021), 6MP (*P* = 0.020), and 6MP+5% (*P* = 0.03) compared with 40%PPO (intensity effect). The decrease in RFR (time effect; comparison with the first time point, 5 s) manifested at 60 s for 6MP+5% (*P* = 0.012) and at 90 s for both 6MP-5% (*P* = 0.016) and 6MP (*P* = 0.016). At task failure, the reduction of RFR was similar (*P* = 0.012) between trials (6MP-5%: 45 ± 21%, 6MP: 37 ± 11%, and 6MP+5%: 37 ± 13%).

Immediately after exhaustive exercise, there were significant effects for time (*P* < 0.001), intensity (*P* < 0.001), and intensity × time interaction (*P* < 0.001) for RFD and RFR in isometric double-twitches (Post; 6MP-5%, 6MP, and 6MP+5%). The decline was higher for potentiated twitch compared to unpotentiated twitch (38–51 vs. ∼30–40%; Table 1).

### Exercise Intensity and Electro-Mechanical Delay

The determination of EMD during dynamic exercise exhibited some variability between time-points, and no changes were evident in either exercise condition (Figure 3B). Based on the variability between time-points, the limit of detection was found to be approximately ± 10% based on a post-study power analysis. For EMD, there was a significant effect for time (*P* < 0.001), intensity (*P* < 0.001), and intensity × time interaction (*P* = 0.007). EMD was increased during 6MP and 6MP+5% exercises (time effect; comparison with the first time point, 5 s), after 270 s (*P* = 0.032) and 120 s (*P* = 0.045) of exercise, respectively.

For unpotentiated twitch, there was no significant effect for time (*P* < 0.557), intensity (*P* < 0.613), and intensity × time interaction (*P* = 0.934). In contrast, for potentiated twitch, we observed a significant effect for time (*P* < 0.001), intensity (*P* = 0.042), and intensity × time interaction (*P* = 0.027). No significant differences were found between baseline and Pre values for EMD during unpotentiated (*P* > 0.629) and potentiated (*P* > 0.575) evoked twitches (Table 1), indicating no effect of the 4 min unloaded exercise on this parameter. The 40%PPO exercise had no significant effect on unpotentiated (*P* > 0.987) and potentiated (*P* > 0.951) twitches EMD (Post *vs*. Pre and baseline). Unlike unpotentiated twitch, the EMD measured during potentiated twitch was significantly elongated after exhaustive exercise (6MP-5%, *P* = 0.003; 6MP, *P* < 0.001; 6MP+5%, *P* = 0.001; Post vs. Pre). The extent of EMD elongation measured during potentiated twitch was lower than during dynamic exercise (∼10 *vs*. ∼15–30% for 6MP-5%, 6MP, and 6MP+5%).

### M-Wave Characteristics

The characteristics of first phase, second phase, and full M-wave of the m. vastus lateralis were determined at the beginning and at the end of loaded dynamic exercise by a single electrical stimulation (Table 2). M-waves could not be determined from the stimulation doublets utilized during exercise, where the aim was to achieve a sufficient force response. None of the measured first phase, second phase, or full M-wave characteristics was affected by the different exercise protocols (end *vs*. start exercise). For amplitude in first phase, second phase, and full M-wave, no significant time effect (*P* = 0.106, *P* = 0.951, and *P* = 0.185, respectively), intensity effect (*P* = 0.845, *P* = 0.539, *P* = 0.433, respectively), and intensity × time interaction effect (*P* = 0.869, *P* = 0.992, *P* = 0.898, respectively) were observed. No significant effects were noted in first phase, second phase, and full M-wave duration for time (*P* = 0.270, *P* = 0.634, and *P* = 0.439, respectively), intensity (*P* = 0.314, *P* = 0.629, and *P* = 0.388, respectively), and intensity × time interaction (*P* = 0.504, *P* = 0.482, *P* = 0.283, respectively). The linear mixed-model analysis of end *vs*. start exercise first phase, second phase, and full M-wave area revealed no significant effects for time (*P* = 0.821, *P* = 0.976, and *P* = 0.854, respectively), intensity (*P* = 0.414, *P* = 0.793, *P* = 0.708, respectively), and intensity × time interaction (*P* = 0.473, *P* = 0.747, *P* = 0.844, respectively).

**TABLE 2:** Effects of dynamic exercise intensity on M-wave parameters.

**Exercise intensity**	**40% PPO**	**6MP-5%**	**6MP**	**6MP+5%**
**First phase**				
***Amplitude (mV)***				
Start exercise	4.2 ± 2.1	3.8 ± 2.8	3.7 ± 1.6	4.1 ± 2.3
End exercise	4.3 ± 2.1	4.5 ± 2.8	4.4 ± 2.8	4.5 ± 3.0
***Duration (ms)***				
Start exercise	8.7 ± 1.8	7.6 ± 1.6	8.0 ± 2.4	7.9 ± 2.1
End exercise	7.9 ± 1.9	8.0 ± 2.5	7.8 ± 3.4	6.7 ± 1.8
***Area (mV ms)***				
Start exercise	32.6 ± 11.5	26.9 ± 19.7	28.2 ± 14.8	29.7 ± 15.9
End exercise	29.0 ± 11.0	29.7 ± 17.8	32.1 ± 19.1	25.2 ± 15.9
**Second phase**				
***Amplitude (mV)***				
Start exercise	5.6 ± 2.1	5.9 ± 1.6	6.1 ± 2.4	6.2 ± 2.6
End exercise	5.6 ± 1.8	6.0 ± 1.4	5.9 ± 2.0	6.3 ± 2.3
***Duration (ms)***				
Start exercise	30.7 ± 9.3	29.6 ± 6.3	29.2 ± 5.7	30.2 ± 5.4
End exercise	29.7 ± 6.8	30.6 ± 7.8	29.5 ± 7.6	27.4 ± 8.2
***Area (mV ms)***				
Start exercise	46.5 ± 18.0	49.5 ± 13.8	51.2 ± 16.5	45.0 ± 19.6
End exercise	46.6 ± 18.7	48.3 ± 9.7	48.0 ± 15.9	49.1 ± 18.6
**Whole M-wave**				
***Amplitude (mV)***				
Start exercise	9.8 ± 2.8	9.6 ± 3.5	9.8 ± 3.4	10.2 ± 3.2
End exercise	9.9 ± 2.9	10.4 ± 3.6	10.4 ± 3.8	10.9 ± 3.3
***Duration (ms)***				
Start exercise	39.5 ± 9.3	37.3 ± 6.0	37.2 ± 5.8	38.1 ± 5.1
End exercise	37.6 ± 5.5	38.6 ± 7.4	37.3 ± 9.0	34.1 ± 7.4
***Area (mV ms)***				
Start exercise	79.2 ± 20.7	76.4 ± 27.8	79.4 ± 21.9	74.8 ± 23.2
End exercise	75.7 ± 19.3	77.9 ± 20.6	80.0 ± 29.6	74.3 ± 27.4

*A single twitch was elicited 15 s after the beginning of exercise at a target intensity (start exercise) and at the end (12 min for 40%PPO and at task failure for 6MP-5%, 6MP, and 6MP+5%). Peak-to-peak amplitude, duration, and area of first phase and second phase and whole M-wave were measured. Data as mean ± SD.*

### Recovery

**FIGURE 4:**
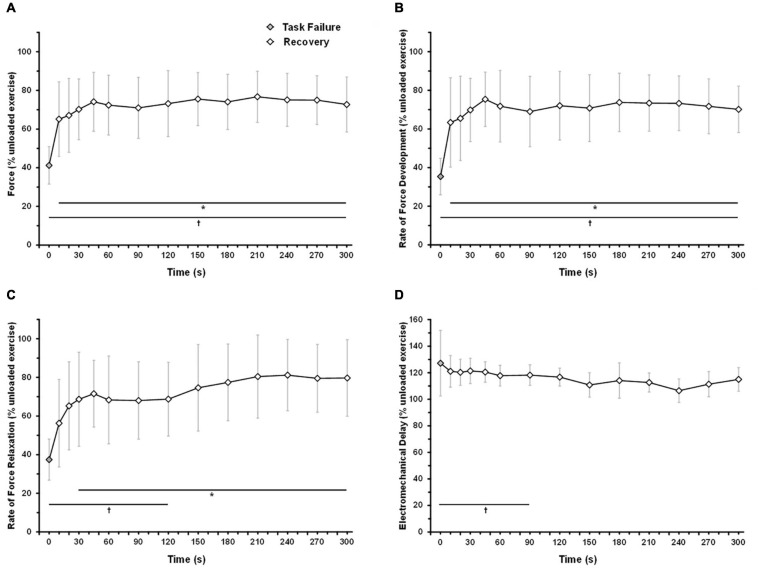
Time-course of force and intratwitch indices recovery following an exhaustive 6-min exercise. The subjects performed one-legged knee-extension exercise at 6MP until exhaustion. At task failure, the load was removed, and the subjects were instructed to maintain the kicking frequency at 60 rpm (unloaded exercise). Force response from VL was elicited by an electrical stimulation (double twitch, 10-ms interval) 10, 20, 30, 45, and 60 s after task failure and subsequently every 30 s until 5-min recovery. **(A)** Force response, **(B)** rate of force development, **(C)** rate of force relaxation, **(D)** electromechanical delay. The data are mean ± SD. Holm–Sidak corrected *post hoc* test results. *CP+5% significantly different from task failure (*P* < 0.05); ^†^CP significantly different from unloaded exercise (*P* < 0.05).

The force and intratwitch indexes of contractile properties were monitored during early recovery from an exhaustive 6MP exercise (Figure 4). A linear mixed-model analysis showed significant effects of time (comparison with task failure) for force (*P* < 0.001), RFD (*P* < 0.001), RFR (*P* < 0.001), and EMD (*P* = 0.008). At only 10 s after task failure, elicited force was significantly higher than at end exercise (65.2 ± 19.3 vs. 41.2 ± 9.7% pre-exercise value, *P* = 0.034; Figure 4A). After 30 s of recovery, the force reached a plateau at ∼70–75% of pre-exercise level. At 5 min after task failure, the force was significantly lower than at pre-exercise level (*P* = 0.005). As illustrated in Figures 4B,C, RFD recovered faster than RFR after task failure. RFD was significantly different from task failure value after only 10 s of recovery (63.4 ± 23.1 vs. 35.4 ± 9.5% pre-exercise value, *P* = 0.018; Figure 4B), while RFR was significantly different from task failure value only after 30 s of recovery (68.7 ± 24.3 vs. 37.4 ± 10.6% pre-exercise value, *P* = 0.047; Figure 4C). A plateau was observed for RFD and RFR from 30 s to 5 min of recovery, corresponding to ∼70% of the pre-exercise level. From 150 s of recovery, RFR was not significantly different from that of the pre-exercise level (*P* = 0.349). EMD remained not significantly different from task failure throughout recovery (*P* > 0.153). From 120 s after task failure, EMD was not significantly different from that of the pre-exercise level (*P* > 0.064).

## Discussion

This *in vivo* study provides a detailed time-course assessment of muscular fatigue development during ongoing exercise in humans, with a special focus on the effects of exhaustive exercise on important contractile characteristics and recovery. With the applied methodology and analyses, we explore how the rate of force development as well as muscle relaxation are reduced in an intensity-dependent manner and how the most intense exhaustive exercise bouts provoke a prolonged electromechanical delay, without altering the measures of excitability assessed *via* percutaneous electrical stimulation.

### Twitch Characteristics

The current observations demonstrate that muscle twitch characteristics are highly dependent on the degree of exercise intensity. The rate of force development and the rate of relaxation are compromised at a fast rate in the initial few minutes followed by a slower gradual reduction. Moreover, the compromised contractile function occurs more rapidly as the intensity is increased. Based on the large body of literature dealing with *in vitro* muscle fatigue models, some hypotheses can be formulated. The time-course decline in RFD and RFR closely resembles the pattern of creatine phosphate hydrolysis, but not metabolic acidosis, during exercise as investigated by ^31^P-MRS (Jones et al., 2008). In agreement, Degroot et al. (1993) have shown that muscle fatigue can develop without meaningful changes in pH, suggesting factors other than acidosis to explain the decline in force-generating capacity. In this respect, the deleterious effects of P_i_ accumulation on muscle function have received increasing attention over the last two decades (Fryer et al., 1995; Fitts, 2008; Nelson and Fitts, 2014; Debold et al., 2016). P_i_ inhibits force by accelerating myosin detachment from actin *via* a decreased number of strongly bound cross-bridges (Debold et al., 2004). P_i_ can also indirectly alter the force-generating capacity of skeletal muscle by reducing Ca^2+^ release by SR. A current hypothesis is that P_i_ can enter the SR and precipitates with Ca^2+^, thereby reducing the amount of free Ca^2+^ released during fatigue (Fitts, 2008). Thus, we propose that our current observations point to P_i_ accumulation to be of primary importance for fatigue development during intense exercise.

Slowing of relaxation is a well-known feature of skeletal muscle fatigue (Gollnick et al., 1991; Hill et al., 2001). A reduction in calcium reuptake by the sarcoplasmic reticulum or reduced kinetics of the cross-bridge dissociation may explain the slowing of relaxation *via* accumulation of ATP hydrolysis by-products such as H^+^ and P_i_ (Palmer et al., 1991; Allen et al., 1995, 2008; Li et al., 2002). It has been shown that acidosis and P_i_ accumulation can alter the cross-bridge dissociation rate (Cady et al., 1989; Palmer et al., 1991; Fitts, 2008). In contrast to RFD, the relationship between slowing of relaxation and fatigue is less evident since prolongation of muscle relaxation can attenuate the force decline between two motor unit recruitments. Such a mechanism, although being beneficial during prolonged isometric contraction, seems detrimental during dynamic exercise when muscle contraction and relaxation alternate at a high frequency (Allen et al., 2008).

The present model allows monitoring of force and intratwitch index recovery early after task failure. Notably, most of the force recovery occurs within the first 30 s following a 6-min exhaustive task. From a methodological perspective, in agreement with previous data (Froyd et al., 2013; Carroll et al., 2017), the present study confirms that the time-course of force measurement after exercise is a crucial issue to assess both the extent of intramuscular muscle fatigue at task failure and during early recovery. Surprisingly, the different intratwitch indices display distinctive recovery profiles. RFD recovers more rapidly than RFR, suggesting that the exercise-induced alteration of SR-Ca^2+^ release recovers quickly than SR-Ca^2+^ sequestration.

### Muscle Excitability Indexes

The M-wave amplitude duration as well as M-wave first and second components remained constant during intense dynamic exercise, indicating that membrane excitability was unaffected.

Action potential triggering and propagation depend on the electrochemical gradient of Na^+^ and K^+^ across the sarcolemma. While it is well established that intense dynamic exercise is characterized by a large disturbance in the intra- and extracellular concentration of electrolytes (McKenna et al., 2008), the implication of altered membrane excitability for skeletal muscle fatigue remains an unresolved issue. Recently, it has been argued that the interpretation of M-wave characteristics is dependent on the analysis of distinct parts of the recorded signal (Rodriguez-Falces and Place, 2017). Thus, we analyzed both the first and second phases of the M-wave. This analysis confirmed that the M-wave characteristics remained constant during exhaustive dynamic exercise. Therefore, the EMD elongation that we observed must be ascribed to compromised EC coupling. During recovery, EMD elongation persists at least 90 s following task failure, whereas force has already recovered for 1 min. As discussed above, this strengthens the concept that muscle excitability is not critically involved in muscle fatigue development nor a crucial issue for recovery from dynamic exercise-induced muscle fatigue.

### Limitations

In the present study, comparisons during dynamic exercise were made using an absolute, rather than relative, timescale. It could be speculated that the expression of data on a relative timescale could provide additional insights. However, absolute expression provides an unbiased representation of the temporal alteration in muscle contractile properties.

Within the current study, we acknowledge that only VL contractile properties are thought to be monitored whereas the whole quadriceps exercises. From a methodological perspective, we used direct percutaneous stimulation of the VL (Burnley, 2009; Casuso et al., 2014) rather than the femoral nerve (Place et al., 2007; Froyd et al., 2013; Black et al., 2017). Indeed we needed to make a compromise between the level of stimulation (electrical intensity and recruited volume mass) and the disturbance of the kick movement during dynamic exercise. At high-intensity exercise, preliminary tests have shown that femoral nerve stimulation induces a greater disturbance in the kicking movement than percutaneous VL stimulation. Due to higher muscle mass recruitment (whole quadriceps *vs*. VL solely), it is difficult for the subject to recover a 60-kick/min rate of kicking after an evoked twitch response induced by femoral nerve stimulation.

Another issue is that only VL fatigue is assumed to be measured throughout the study. Although electrode placement was optimized to stimulate preferably VL, a crosstalk from nearby quadriceps parts (rectus femoris, vastus medial, and vastus intermedius) is highly plausible. Since the electrode position was maintained throughout the protocol and replicated from trial to trial and the changes in twitch responses were normalized to “initial twitch,” the influence is expected to be similar across trials. Additionally, it has been shown that, in such a setting, VL fatigue constitutes a reliable surrogate measure of whole quadriceps fatigue using voluntary or electrically evoked contractions (Rutherford et al., 1986; Behm et al., 1996; Martin et al., 2004; Place et al., 2007).

Another limitation is that the contractile properties of skeletal muscle are not only altered by fatigue. It has been shown that RFD may increase as a result of post-activation potentiation (Rassier and Macintosh, 2000; Hodgson et al., 2005), i.e., the previous activation of a muscle may lead to increased RFD. Accordingly, the contractile properties are the result of a complex interplay between fatigue and potentiation. Within the current investigation, this may explain the two- to fourfold difference in RFD and RFR values between “dynamic” and “isometric” measurements, that is, during the eccentric–relaxation phase in dynamic exercise *vs*. the isometric–uncontracted state. Further studies are needed to delineate the respective contribution of potentiation and fatigue regarding contractile property alteration in the present model.

### Perspectives

Our study reveals that the intratwitch indexes of contractile properties are useful to monitor intracellular events that occur in the skeletal muscle during dynamic exercise. The muscle contractile properties are compromised in an intensity-dependent manner during exhaustive dynamic exercise in humans. No indication of compromised excitability exists in this setup. The early decline in the rate of force development and relaxation during heavy exercise likely reflects rapid alterations in intracellular Ca^2+^ regulation. Interestingly, the present model may help to fill the gap between *in vivo* human model of fatigue and *in vitro* models, for which an extensive body of literature exists. The potential applications of this new model are numerous and may be of interest for exercise physiologists and clinicians involved in the management of patients experiencing exercise intolerance, such as post-COVID-19 fatigue syndrome. Furthermore, the present model provides a valuable basis to understand the mechanisms that underlie training-induced intramuscular resistance to fatigue and to design interventions targeting to improve endurance capacity.

## Data Availability Statement

The raw data supporting the conclusions of this article will be made available by the authors, without undue reservation.

## Ethics Statement

The studies involving human participants were reviewed and approved by the Copenhagen and Frederiksberg Ethics Committee (H-16035688). The patients/participants provided their written informed consent to participate in this study.

## Author Contributions

FR, LN, JA, and NN contributed to the conception and design of the study, contributed to the acquisition, analysis, and interpretation of data for this work, edited the text and revised it critically for important intellectual content, and approved the final version for submission. FR drafted the manuscript and prepared the figures. All authors contributed to the article and approved the submitted version.

## Conflict of Interest

The authors declare that the research was conducted in the absence of any commercial or financial relationships that could be construed as a potential conflict of interest.

## References

[B1] Allen D. G. Lamb G. D. Westerblad H. (2008). Skeletal muscle fatigue: cellular mechanisms. *Physiol. Rev.* 88 287–332. 10.1152/physrev.00015.2007 18195089

[B2] Allen D. G. Lännergren J. Westerblad H. (1995). Muscle cell function during prolonged activity: cellular mechanisms of fatigue. *Exp. Physiol.* 80 497–527. 10.1113/expphysiol.1995.sp003864 7576593

[B3] Amann M. Venturelli M. Ives S. J. McDaniel J. Layec G. Rossman M. J. (2013). Peripheral fatigue limits endurance exercise via a sensory feedback-mediated reduction in spinal motoneuronal output. *J. Appl. Physiol. Bethesda Md 1985* 115 355–364. 10.1152/japplphysiol.00049.2013 23722705 PMC3743006

[B4] Andersen P. Adams R. P. Sjøgaard G. Thorboe A. Saltin B. (1985). Dynamic knee extension as model for study of isolated exercising muscle in humans. *J. Appl. Physiol. Bethesda Md 1985* 59 1647–1653. 10.1152/jappl.1985.59.5.1647 4066596

[B5] Behm D. G. St-Pierre D. M. Perez D. (1996). Muscle inactivation: assessment of interpolated twitch technique. *J. Appl. Physiol. Bethesda Md 1985* 81 2267–2273. 10.1152/jappl.1996.81.5.2267 8941554

[B6] Bigland-Ritchie B. R. Dawson N. J. Johansson R. S. Lippold O. C. (1986). Reflex origin for the slowing of motoneurone firing rates in fatigue of human voluntary contractions. *J. Physiol.* 379 451–459. 10.1113/jphysiol.1986.sp016263 3560001 PMC1182907

[B7] Black M. I. Jones A. M. Blackwell J. R. Bailey S. J. Wylie L. J. McDonagh S. T. J. (2017). Muscle metabolic and neuromuscular determinants of fatigue during cycling in different exercise intensity domains. *J. Appl. Physiol. Bethesda Md 1985* 122 446–459. 10.1152/japplphysiol.00942.2016 28008101 PMC5429469

[B8] Blackburn J. T. Bell D. R. Norcross M. F. Hudson J. D. Engstrom L. A. (2009). Comparison of hamstring neuromechanical properties between healthy males and females and the influence of musculotendinous stiffness. *J. Electromyogr. Kinesiol. Off. J. Int. Soc. Electrophysiol. Kinesiol.* 19 e362–e369. 10.1016/j.jelekin.2008.08.005 18829346

[B9] Brownstein C. G. Millet G. Y. Thomas K. (2021). Neuromuscular responses to fatiguing locomotor exercise. *Acta Physiol. Oxf. Engl.* 231 e13533. 10.1111/apha.13533 32627930

[B10] Burnley M. (2009). Estimation of critical torque using intermittent isometric maximal voluntary contractions of the quadriceps in humans. *J. Appl. Physiol. Bethesda Md 1985* 106 975–983. 10.1152/japplphysiol.91474.2008 19150854

[B11] Burnley M. Vanhatalo A. Fulford J. Jones A. M. (2010). Similar metabolic perturbations during all-out and constant force exhaustive exercise in humans: a (31)P magnetic resonance spectroscopy study. *Exp. Physiol.* 95 798–807. 10.1113/expphysiol.2010.052688 20360422

[B12] Burnley M. Vanhatalo A. Jones A. M. (2012). Distinct profiles of neuromuscular fatigue during muscle contractions below and above the critical torque in humans. *J. Appl. Physiol. Bethesda Md 1985* 113 215–223. 10.1152/japplphysiol.00022.2012 22556396

[B13] Cady E. B. Jones D. A. Lynn J. Newham D. J. (1989). Changes in force and intracellular metabolites during fatigue of human skeletal muscle. *J. Physiol.* 418 311–325. 10.1113/jphysiol.1989.sp017842 2621621 PMC1189973

[B14] Carroll T. J. Taylor J. L. Gandevia S. C. (2017). Recovery of central and peripheral neuromuscular fatigue after exercise. *J. Appl. Physiol. Bethesda Md 1985* 122 1068–1076. 10.1152/japplphysiol.00775.2016 27932676

[B15] Casuso R. A. Melskens L. Bruhn T. Secher N. H. Nordsborg N. B. (2014). Glucocorticoids improve high-intensity exercise performance in humans. *Eur. J. Appl. Physiol.* 114 419–424. 10.1007/s00421-013-2784-7 24327175

[B16] Cheng A. J. Place N. Westerblad H. (2018). Molecular basis for exercise-induced fatigue: the importance of strictly controlled cellular Ca^2+^ handling. *Cold Spring Harb. Perspect. Med.* 8:a029710. 10.1101/cshperspect.a029710 28432118 PMC5793735

[B17] Debold E. P. Dave H. Fitts R. H. (2004). Fiber type and temperature dependence of inorganic phosphate: implications for fatigue. *Am. J. Physiol. Cell Physiol.* 287 C673–C681. 10.1152/ajpcell.00044.2004 15128502

[B18] Debold E. P. Fitts R. H. Sundberg C. W. Nosek T. M. (2016). Muscle fatigue from the perspective of a single crossbridge. *Med. Sci. Sports Exerc.* 48 2270–2280. 10.1249/MSS.0000000000001047 27434086

[B19] Degroot M. Massie B. M. Boska M. Gober J. Miller R. G. Weiner M. W. (1993). Dissociation of [H+] from fatigue in human muscle detected by high time resolution 31P-NMR. *Muscle Nerve* 16 91–98. 10.1002/mus.880160115 8423837

[B20] Ducrocq G. P. Hureau T. J. Meste O. Blain G. M. (2017). Increased fatigue response to augmented deceptive feedback during cycling time trial. *Med. Sci. Sports Exerc.* 49 1541–1551. 10.1249/MSS.0000000000001272 28319585

[B21] Fitts R. H. (2008). The cross-bridge cycle and skeletal muscle fatigue. *J. Appl. Physiol. Bethesda Md 1985* 104 551–558. 10.1152/japplphysiol.01200.2007 18162480

[B22] Fletcher W. M. (1902). The relation of oxygen to the survival metabolism of muscle. *J. Physiol.* 28 474–498. 10.1113/jphysiol.1902.sp000930 16992637 PMC1540599

[B23] Froyd C. Millet G. Y. Noakes T. D. (2013). The development of peripheral fatigue and short-term recovery during self-paced high-intensity exercise. *J. Physiol.* 591 1339–1346. 10.1113/jphysiol.2012.245316 23230235 PMC3607875

[B24] Fryer M. W. Owen V. J. Lamb G. D. Stephenson D. G. (1995). Effects of creatine phosphate and P(i) on Ca2+ movements and tension development in rat skinned skeletal muscle fibres. *J. Physiol.* 482(Pt 1) 123–140. 10.1113/jphysiol.1995.sp020504 7730977 PMC1157758

[B25] Gollnick P. D. Körge P. Karpakka J. Saltin B. (1991). Elongation of skeletal muscle relaxation during exercise is linked to reduced calcium uptake by the sarcoplasmic reticulum in man. *Acta Physiol. Scand.* 142 135–136. 10.1111/j.1748-1716.1991.tb09139.x 1831584

[B26] Hill C. A. Thompson M. W. Ruell P. A. Thom J. M. White M. J. (2001). Sarcoplasmic reticulum function and muscle contractile character following fatiguing exercise in humans. *J. Physiol.* 531 871–878. 10.1111/j.1469-7793.2001.0871h.x 11251066 PMC2278486

[B27] Hodgson M. Docherty D. Robbins D. (2005). Post-activation potentiation: underlying physiology and implications for motor performance. *Sports Med. Auckl. NZ* 35 585–595. 10.2165/00007256-200535070-00004 16026172

[B28] Hostrup M. Bangsbo J. (2017). Limitations in intense exercise performance of athletes - effect of speed endurance training on ion handling and fatigue development. *J. Physiol.* 595 2897–2913. 10.1113/JP273218 27673449 PMC5407979

[B29] Jones A. M. Wilkerson D. P. DiMenna F. Fulford J. Poole D. C. (2008). Muscle metabolic responses to exercise above and below the “critical power” assessed using 31P-MRS. *Am. J. Physiol. Regul. Integr. Comp. Physiol.* 294 R585–R593. 10.1152/ajpregu.00731.2007 18056980

[B30] Krüger R. L. Aboodarda S. J. Jaimes L. M. MacIntosh B. R. Samozino P. Millet G. Y. (2019). Fatigue and recovery measured with dynamic properties versus isometric force: effects of exercise intensity. *J. Exp. Biol.* 222(Pt 9) jeb197483. 10.1242/jeb.197483 30890621

[B31] Li J. L. Wang X. N. Fraser S. F. Carey M. F. Wrigley T. V. McKenna M. J. (2002). Effects of fatigue and training on sarcoplasmic reticulum Ca(2+) regulation in human skeletal muscle. *J. Appl. Physiol. Bethesda Md 1985* 92 912–922. 10.1152/japplphysiol.00643.2000 11842021

[B32] Martin V. Millet G. Y. Martin A. Deley G. Lattier G. (2004). Assessment of low-frequency fatigue with two methods of electrical stimulation. *J. Appl. Physiol. Bethesda Md 1985* 97 1923–1929. 10.1152/japplphysiol.00376.2004 15258127

[B33] McKenna M. J. Bangsbo J. Renaud J.-M. (2008). Muscle K+, Na+, and Cl disturbances and Na+-K+ pump inactivation: implications for fatigue. *J. Appl. Physiol. Bethesda Md 1985* 104 288–295. 10.1152/japplphysiol.01037.2007 17962569

[B34] Millet G. Y. Millet G. P. Lattier G. Maffiuletti N. A. Candau R. (2003). Alteration of neuromuscular function after a prolonged road cycling race. *Int. J. Sports Med.* 24 190–194. 10.1055/s-2003-39088 12740737

[B35] Nelson C. R. Fitts R. H. (2014). Effects of low cell pH and elevated inorganic phosphate on the pCa-force relationship in single muscle fibers at near-physiological temperatures. *Am. J. Physiol. Cell Physiol.* 306 C670–C678. 10.1152/ajpcell.00347.2013 24452378

[B36] Neyroud D. Cheng A. J. Bourdillon N. Kayser B. Place N. Westerblad H. (2016). Muscle fatigue affects the interpolated twitch technique when assessed using electrically-induced contractions in human and rat muscles. *Front. Physiol.* 7:252. 10.3389/fphys.2016.00252 27445844 PMC4924481

[B37] Olsson K. Cheng A. J. Al-Ameri M. Wyckelsma V. L. Rullman E. Westerblad H. (2020). Impaired sarcoplasmic reticulum Ca2+ release is the major cause of fatigue-induced force loss in intact single fibres from human intercostal muscle. *J. Physiol.* 598 773–787. 10.1113/JP279090 31785106

[B38] Palmer R. E. Simnett S. J. Mulligan I. P. Ashley C. C. (1991). Skeletal muscle relaxation with diazo-2: the effect of altered pH. *Biochem. Biophys. Res. Commun.* 181 1337–1342. 10.1016/0006-291x(91)92085-x 1764084

[B39] Place N. Maffiuletti N. A. Martin A. Lepers R. (2007). Assessment of the reliability of central and peripheral fatigue after sustained maximal voluntary contraction of the quadriceps muscle. *Muscle Nerve* 35 486–495. 10.1002/mus.20714 17221875

[B40] Rannou F. Nybo L. Andersen J. E. Nordsborg N. B. (2019). Monitoring muscle fatigue progression during dynamic exercise. *Med. Sci. Sports Exerc.* 51 1498–1505. 10.1249/MSS.0000000000001921 30741747

[B41] Rassier D. E. Macintosh B. R. (2000). Coexistence of potentiation and fatigue in skeletal muscle. *Braz. J. Med. Biol. Res. Rev. Bras. Pesqui. Medicas E Biol.* 33 499–508. 10.1590/s0100-879x2000000500003 10775880

[B42] Reid C. (1927). The mechanism of voluntary muscular fatigue. *Br. Med. J.* 2 545–546. 10.1136/bmj.2.3481.545 20773416 PMC2524887

[B43] Rodriguez-Falces J. Place N. (2017). Muscle excitability during sustained maximal voluntary contractions by a separate analysis of the M-wave phases. *Scand. J. Med. Sci. Sports* 27 1761–1775. 10.1111/sms.12819 28028847

[B44] Rutherford O. M. Jones D. A. Newham D. J. (1986). Clinical and experimental application of the percutaneous twitch superimposition technique for the study of human muscle activation. *J. Neurol. Neurosurg. Psychiatry* 49 1288–1291. 10.1136/jnnp.49.11.1288 3794735 PMC1029078

[B45] Vanhatalo A. Black M. I. DiMenna F. J. Blackwell J. R. Schmidt J. F. Thompson C. (2016). The mechanistic bases of the power-time relationship: muscle metabolic responses and relationships to muscle fibre type. *J. Physiol.* 594 4407–4423. 10.1113/JP271879 26940850 PMC4967754

[B46] Westerblad H. Lännergren J. (1991). Slowing of relaxation during fatigue in single mouse muscle fibres. *J. Physiol.* 434 323–336. 10.1113/jphysiol.1991.sp018472 1902516 PMC1181420

